# AURKA induces EMT by regulating histone modification through Wnt/β-catenin and PI3K/Akt signaling pathway in gastric cancer

**DOI:** 10.18632/oncotarget.8888

**Published:** 2016-04-21

**Authors:** Xi Liu, Zhaoxia Li, Yue Song, Rui Wang, Lei Han, Qixue Wang, Kui Jiang, Chunsheng Kang, Qingyu Zhang

**Affiliations:** ^1^ Department of Gastroenterology, Tianjin Medical University General Hospital, Tianjin 300052, China; ^2^ Department of Neurosurgery, Tianjin Medical University General Hospital, Heping District, Tianjin 300052, China; ^3^ Laboratory of Neuro-Oncology, Tianjin Neurological Institute, Tianjin 300052, China

**Keywords:** AURKA, Wnt/β-catenin pathway, PI3K/Akt pathway, histone modification

## Abstract

Gastric cancer, a highly invasive and aggressive malignancy, is the third leading cause of death from cancer worldwide. Genetic association studies have successfully revealed several important genes consistently associated with gastric cancer to date. However, these robust gastric cancer-associated genes do not fully elucidate the mechanisms underlying the development and progression of the disease. In the present study, we performed an alternative approach, a gene expression-based genome-wide association study (eGWAS) across 13 independent microarray experiments (including 251 gastric cancer cases and 428 controls), to identify top candidates (p<0.00001). Additionally, we conducted gene ontology analysis, pathway analysis and network analysis and identified aurora kinase A (AURKA) as our candidate. We observed that MLN8237, which is a specific inhibitor of AURKA, decreased the β-catenin and the phosphorylation of Akt1 and GSK-3β, as well as blocked the Akt and Wnt signaling pathways. Furthermore, MLN8237 arrested the cells in the G2/M phase. The activity of Wnt and Akt signaling pathways affected the level of histone methylation significantly, and we supposed that MLN8237 affected the level of histone methylation through these two signaling pathways. Additionally, the treatment of MLN8237 influenced the level of H3K4 me1/2/3 and H3K27 me1/2/3. Chip data on cell lines suggested that MLN8237 increases the level of H3K27 me3 on the promoter of Twist and inhibits EMT (epithelial-mesenchymal transition). In summary, AURKA is a potential therapeutic target in gastric cancer and induces EMT through histone methylation.

## INTRODUCTION

Gastric cancer is a highly invasive and aggressive malignancy and one of the leading causes of death from cancer worldwide; therefore, investigations into its initiation and development are of great importance [1–3]. The EMT is important during carcinogenesis and metastatic progression, and this term has been more liberally referred as a recognizable change in cellular phenotype characterized by the loss of cell junctions and gain of migratory behaviors. Recent studies have established that aberrant EMT activation in the human stomach is closely associated with gastric carcinogenesis and tumor progression [4, 5]. The prognosis of gastric cancer patients usually depends on the early detection and treatment of malignant tumor characteristics such as invasion and metastasis, which are the primary causes of treatment failure. Therefore, the exploration of gastric cancer initiation and progression mechanisms may improve early diagnosis and treatment efficacy.

In the past years, numerous genome-wide association studies (GWASs) have revealed significant associations of single-nucleotide polymorphisms SNPs with susceptibility to gastric cancer and have refreshed the genetic knowledge of gastric cancer [6–10]. Recently, there were several studies aimed to discover candidate (SNPs) for gastric cancer, such as SNPs at 1q22, 10q23 and 5p13.1 [11–14]. However, there have been few fully identified, functionally important genes in the pathogenesis of gastric cancer because many genes are often detected as significant in each microarray experiment, and it is difficult to subselect optimal candidates from individual studies for further verification.

Our hypothesis is that those genes most repeatedly implicated across a large set of experimental representations of gastric cancer can serve as data-driven causal gastric cancer genes and candidates for validation. The method is viable because many of these sources are publicly available; we selected 13 independent microarray experiments for gastric cancer. The identification was followed by confirmatory functional studies using assays *in vitro* and mouse models.

A wealth of epigenomic data has identified abnormal regulation of epigenetic processes as a prominent theme. Recurrent somatic alterations involved in DNA methylation, post-translational histone modification and chromatin remodeling have highlighted the importance of the epigenetic regulation of gene expression in the initiation and maintenance of various malignancies [15–18]. However, the mechanisms of malignant transformation driven by aberrant epigenetic regulators require a thorough understanding. In the present study, we identified the most significant candidate gene from gastric cancer and normal gastric mucosa and researched the mechanisms underlying the initiation of gastric cancer, and identified the aberrant epigenetic regulations. This study offers the opportunity to gain insight into key genes, key pathways and nodes of epigenetic regulation, further enhancing our ability to deliver effective novel compounds for clinical target therapy.

## RESULTS

### eGWASs identify AURKA as a functional candidate gene for gastric cancer

**Figure 1:**
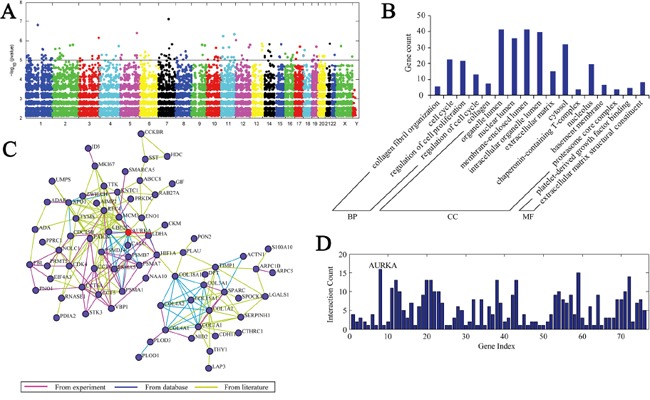
**A.** eGWAS for gastric cancer using a Fisher's exact test. Plot of –log10(*P* value)(*y* axis) by chromosomal position(*x* axis). *P* values for each gene were calculated from our eGWAS across 13 microarray experiments with 679 gastric cancer case-control microarray samples(251 cases and 428 controls). The red line indicates *P* =0.00001, and there were 184 genes *P*<0.00001. **B.** These 184 differential genes were categorized into three groups—biological process, cellular component and molecular function—through gene ontology analysis. The results showed that there was enrichment in the cell cycle and collagen. **C. D.** Network analysis demonstrated that AURKA is the center of the network and consisted of 184 differential genes.

We performed eGWASs for gastric cancer using 13 independent microarray experiments, and 679 samples were collected from public repositories. Additionally, we ranked all 30,663 genes by the likelihood that repeated differential expression for that gene was due to chance, and then controlled for Fisher's exact test. To overview which molecular functions were most shared in the highest ranked genes in our gastric cancer eGWAS, we took 184 genes (Bonferroni threshold, *P*<1.0×10^−5^) from our eGWAS (Figure 1A, Table S1). Next, we estimated the enrichment of Gene Ontology (GO) terms. Interestingly, “cell cycle” and “collagen” functions were the most implicated of the top-ranked genes (Figure 1B). We then performed KEGG pathway analysis and calculated the enrichment *P*-value (Table S2). However, there was no implicated enrichment in any signaling pathway. Finally, we conducted network analysis, which integrated the experiments, database and literature, and the results demonstrated that AURKA is the center of the network (Figure 1C, 1D).

Our gastric cancer candidate gene, AURKA (Figure 1; Fisher's exact test; P=6.83×10^−6^), was markedly differentially expressed in experiments studying gastric cancer with normal gastric mucosa. AURKA is located on chromosome 20q13 and encodes a centrosome-associated, cell cycle-regulated serine/threonine kinase involved in mitosis [22]. Previous studies have revealed that the overexpression of AURKA results in centrosome amplification and cytokinesis failure, causing aneuploidy [23]. Furthermore, AURKA regulates several important proteins such as AKT, β-catenin and p53 in cancer cells [24–28]. The findings suggest that AURKA might stimulate the generation of gastric cancer.

### MLN8237 decreased the phosphorylation of p-Akt1 p-GSK-3β and β-catenin and suppressed cell viability and invasive growth

**Figure 2:**
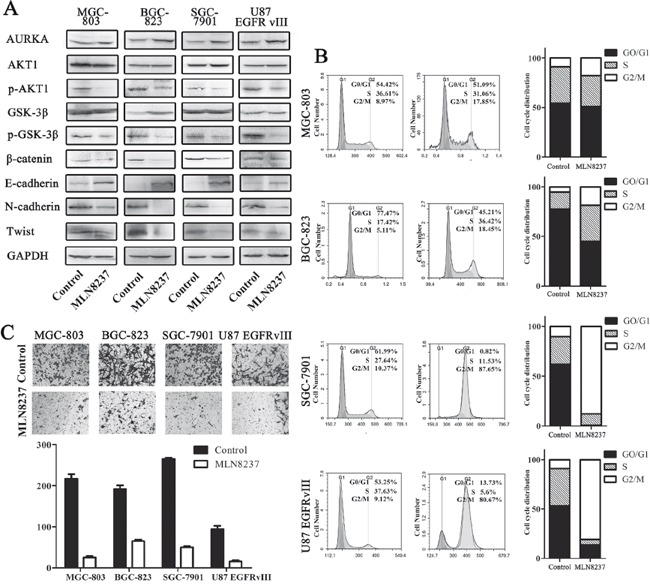
MLN8237 blocked the Wnt and Akt signaling pathways and induced the arrest of cells in the G2/M phase **A.** β-catenin and related target protein expression were assessed by Western blot analysis. GAPDH was, respectively, used as the total protein loading control. **B.** The cell cycle distributions were detected by flow cytometry. The data revealed a significantly increased percentage of cells in the G2/M phase in all four cell lines. **C.** Transwell assays for assessing the invasion of the MLN8237-treated cells(P<0.01).

To validate the effect of MLN8237 on gastric cancer and glioblastoma cell lines, Western blotting results demonstrated the reduced expression of β-catenin and phosphorylation of p-AKT1 and p-GSK-3β. However, the expression of AURKA was slightly increased. These results confirmed that MLN8237 suppressed the activity of the Wnt/β-catenin and PI3K/Akt signaling pathways (Figure 2A). Furthermore, to determine whether the treatment of MLN8237 was accompanied by a change of EMT, we detected the expression of E-cadherin, N-cadherin and Twist following MLN8237 treatment (Figure 2A). The expression of E-cadherin was increased, the expression of N-cadherin and Twist was decreased in the MLN8237-treated cells.

**Figure 3:**
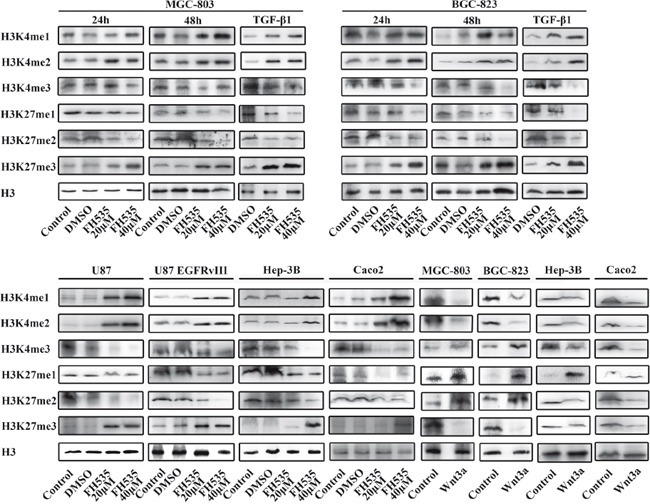
The Wnt/β-catenin signaling pathway regulates histone methylation The expression levels of H3K4 me1/me2/me3 and H3K27 me1/me2/me3 were assessed by Western blot analysis. Histone H3 was used as the nuclear extraction loading control. Treatment with FH535(20μM and 40μM) and Wnt3a(100 ng/ml) regulated the Wnt/β-catenin signaling pathway, and TGF(100 ng/ml) induced the process of EMT.

The cell viability of MGC-803, BGC-823, SGC-7901 and U87 EGFRvIII cell lines was measured by calculating the cell cycle distribution following MLN8237 treatment (Figure 2B). As shown in Figure 2B, the G0/G1 phase fractions of the control MGC-803, BGC-823, SGC-7901 and U87 EGFRvIII cells were 54.42%, 77.47%, 61.99% and 53.25%, respectively. However, treatment with MLN8237decreased the percentage of cells in the G0/G1 phase to 51.09%, 45.21%, 0.82% and 13.73%, respectively. Additionally, the G2/M phase fractions in the control cells were 8.97%, 5.11%, 10.37% and 9.12%, respectively. However, MLN8237 treatment increased the percentage of cells in the G2/M phase to 17.85%, 18.45%, 87.65% and 80.67%, respectively. The results suggested that MLN8237 treatment arrested the cells in the G2/M phase. As shown in Figure 3C, treatment with MLN8237 significantly suppressed the invasion of cells compared with that of the control group (P<0.01).

### The Wnt/β-catenin signaling pathway regulates the methylation and acetylation of H3K4 and H3K27

Above study have demonstrated a significant decrease in β-catenin and the phosphorylation of GSK-3β following MLN8237 treatment. AURKA was shown to increased β-catenin and phosphorylation of GSK-3β, as well as the activity of the Wnt/β-catenin signaling pathway. To investigate the role of the Wnt/β-catenin signaling pathway in regulating histone methylation, we performed Western blot to analyze the change in histone methylation following treatment with FH535 and Wnt3a (Figure 3).

FH535 is a small-molecule inhibitor of the β-catenin/TCF4 complex. In MGC-803 and BGC-823 gastric cancer cells, the levels of H3K4 me1/me2 and H3K27 me3 were increased, those of H3K4 me3 and H3K27 me1/2 were decreased at 20μM FH535 and continued to increase at 40μM FH535 in a dose-dependent manner after FH535 treatment for 24 hours. Furthermore, the changes were consistent with the prolongation of the time of FH535 treatment. Consistent with the findings above, the methylation of H3K4 and H3K27 tended to be uniform in Hep-3B hepatocarcinoma cells, colon cancer Caco2 cells and glioma U87and U87 EGFRvIII cells.

Wnt3a, a canonical Wnt ligand, activates the canonical Wnt/β-catenin signaling pathway. To evaluate whether the change in the methylation of H3K4 and H3K27 was relevant to the status of the Wnt/β-catenin signaling pathway, we enhanced Wnt/β-catenin signaling using exogenous Wnt3a. In MGC-803 and BGC-823 gastric cancer cells, Hep-3B hepatocarcinoma cells and Caco2 colon cancer cells, the levels of H3K4 me1/2 and H3K27 me3 were decreased, and those of H3K4 me3 and H3K27 me1/2 were increased with 100 ng/ml Wnt3a treatment for 24 hours.

TGF-β1 is one of the most well-known inducers of EMT and binds to its receptors on the cell membrane to phosphorylate Smads. We cultured MGC-803 and BGC-823 cells with FH535 at 20 and 40μM following treatment with TGF-β1 for 24 hours. The alterations were evident after EMT was induced by TGF-β1 and were consistent with the results with FH535 treatment only.

### The PI3K/Akt signaling pathway regulates the methylation and acetylation of H3K4 and H3K27

**Figure 4:**
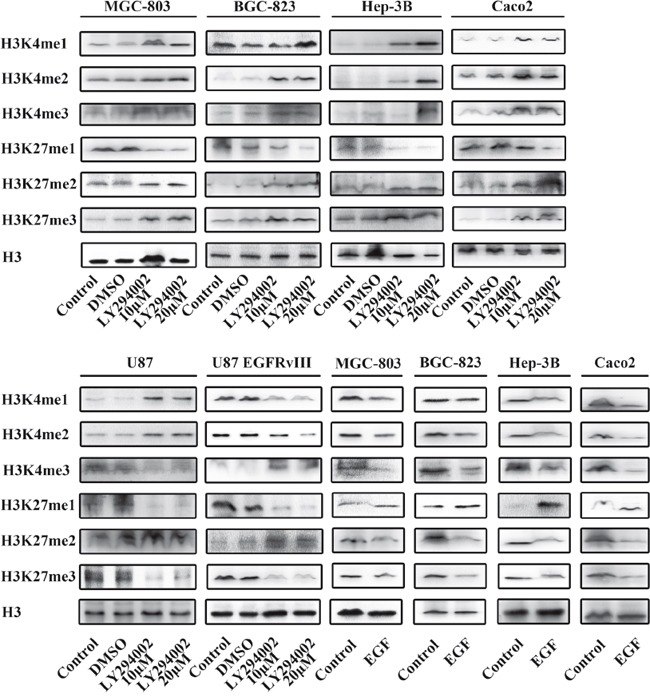
The Akt/PI3K signaling pathway promotes histone methylation Treatment with LY294002(10μM and 20μM) and EGF(100 ng/ml) affected the Akt/PI3K signaling pathway.

The above findings demonstrated a significant decrease in p-Akt1 following MLN8237 treatment. AURKA regulates p-Akt1 and the activity of thePI3K/Akt signaling pathway. To investigate the role of the PI3K/Akt signaling pathway in regulating histone modification, we performed Western blotting to analyze the change in histone methylation following treatment with LY294002 and EGF (Figure 4).

Previous studies have shown that LY294002, which suppresses PI3K, can inhibit the expression of p-Akt in cancer. In MGC-803 and BGC-823 gastric cancer cells, Hep-3B hepatocarcinoma cells and Caco2 colon cancer cells, the levels of H3K4 me1/2/3 and H3K27 me2/3 were increased, and those of H3K27 me1 were decreased following treatment with 10μM LY294002. There was no change noted LY294002 after 20μM treatment. However, in U87 glioma cells, the levels of H3K4 me1/2 and H3K27 me2 were increased, and those of H3K4 me3 and H3K27 me1/3 were decreased following treatment with 10 and 20μM LY294002.

EGF is an upstream activator of the AKT pathway. To evaluate whether the change in the methylation and acetylation of H3K4 and H3K27 was relevant to the status of the PI3K/Akt signaling pathway, we enhanced PI3K/Akt signaling with exogenous EGF. The levels of H3K4 me1/2/3 and H3K27 me2/3 were decreased, and those of H3K27 me1 were increased in MGC-803, BGC-823, Hep-3B and Caco2 cells following treatment with 100 ng/ml EGF.

### MLN8237 regulates the modification of histone and increases the expression of H3K27 me3 in the Twist promoter

**Figure 5:**
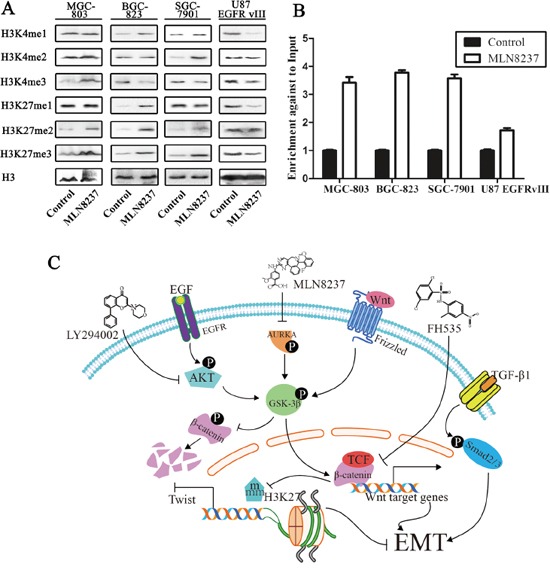
The change in histone methylation of H3K4 and H3K27 is assessed by Western blot analysis **A.** H3 was, respectively, used as the nuclear extraction protein loading control. **B.** ChIP demonstrated that the level of H3K27 me3 in the Twist promoter was increased following MLN8237 treatment (P<0.01). **C.** MLN8237 suppresses the Wnt/β-catenin and PI3K/Akt signaling pathways through inhibition of the phosphorylation of GSK-3β, and inhibition of the EMT process through increasing the level of H3K27 me3 in the Twist promoter.

The results demonstrated an increased expression of H3K27 me2/3 in the gastric cancer cell lines MGC-803, BGC-823 and SGC-7901. However, the expression of H3K4 me1/2/3 and H3K27 me1 showed no consistent tendency following MLN8237 treatment in these gastric cancer cell lines. Furthermore, the results showed decreased expression of H3K4 me1/3 and H3K27 me1/3 in U87 EGFRvIII cells. The effect on histone methylation in gastric cancer cells was very different in U87 EGFRvIII cells (Figure 5A).

To examine the functional role of H3K27 me3 in the promoter region of Twist, we performed ChIP with quantitative PCR analysis (ChIP-qPCR). The results showed that the level of H3K27 me3 in the promoter of Twist increased following MLN8237 treatment (P<0.01, Figure 5B). H3K27 me3 is a repressive histone marker, and the results above suggest that the level of Twist was decreased.

### Inhibition of AURKA suppresses gastric cancer and glioblastoma growth in xenograft models

**Figure 6:**
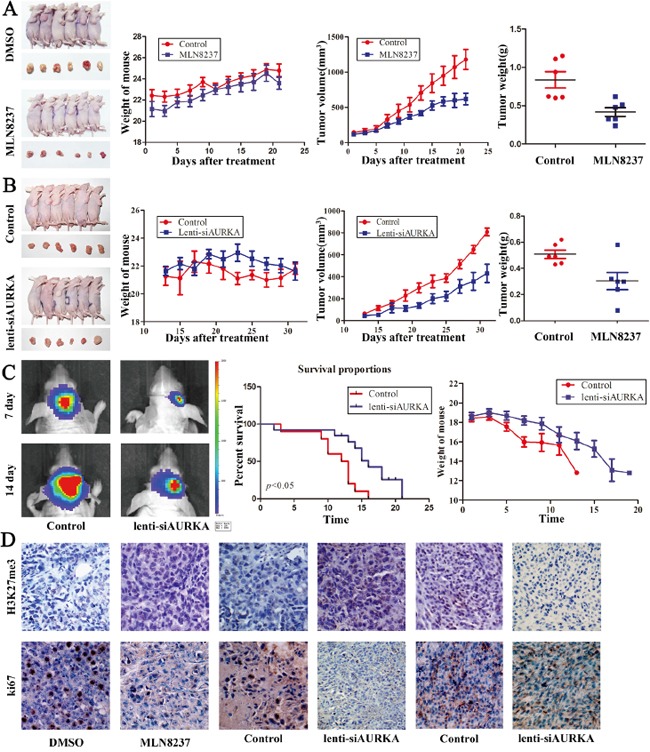
Treatment with MLN8237 and the decreased level of AURKA suppress gastric cancer and glioblastoma growth *in vivo* **A, B.** Mice and tumor samples from the MGC-803/DMSO, MGC-803/MLN8237, MGC-803/Control and MGC-803/lenti-siAURKA groups. The mouse body weights, and tumor growth and weight were evaluated using the *in vivo* proliferation assay. **C.** Bioluminescent images from the control and lenti-siAURKA animals at 14 and 21 days after tumor implantation. Contrasting survival benefits were observed in mice, and mouse body weights were evaluated. **D.** Representative photomicrographs of immunohistochemistry for H3K27 me3 and ki67 on implanted tumor sections.

MLN8237 treatment suppresses gastric cancer and glioma tumorigenesis *in vitro*. To verify the role of AURKA, we established a subcutaneous gastric carcinoma model using MGC-803 cells as described previously. Seven days after implantation, DMSO and MLN8237 were intraperitoneally injected every two days for 21 days. The mouse and tumor weights were measured, and the tumor volume was measured using calipers to measure the tumor length and width (Figure 6A). Treatment with MLN8237 suppressed the growth of gastric cancer cells compared with the DMSO-treated group significantly. Additionally, to confirm the role of AURKA, we established a subcutaneous gastric carcinoma model using the MGC-803 cell line and an orthotopic glioma model using the U87 EGFRvIII cell line. Compared with the Lenti-NC-treated MGC-803 cells, the Lenti-siAURKA-treated tumor was suppressed significantly (Figure 6B). Bioluminescence imaging showed tumor stasis in the Lenti-siAURKA group on day 14, and the results demonstrated prolonged survival by Kaplan-Meier survival curves (*P*<0.05; Figure 6C). In addition, immunohistochemistry was used to compare the H3K27 me3 and Ki67 expression levels in these groups. For cell proliferation markers, Lenti-siAURKA treatment decreased the expression of Ki67. MLN8237 and Lenti-siAURKA treatment increased the expression of H3K27 me3 in gastric carcinoma models (Figure 6D). However, Lenti-siAURKA treatment decreased the expression of H3K27 me3 in the glioblastoma intracranial model, and the change worked together with the result shown in Western blot analysis.

### H3K27 me3 expression accumulates in gastric cancer

Immunostaining analysis revealed H3K27 me3 protein expression in gastric cancer, and the expression levels were significantly greater in gastric adenocarcinoma than in normal and paracarcinoma tissues (Table 1, Figure S1). However, age and gender were not significantly associated with clinical outcome.

**Table 1:** Immunostaining analysis revealed the expression levels of H3K27me3 in gastric adenocarcinoma, paracarcinoma and normal tissues

Variable	−	+	++	+++	Positive rate	Cases	χ^2^value	P value
Gender								
Male	12	36	14	4	81.8%	66	1.844	0.605
Female	9	8	6	3	65.4%	26		
Age, year								
≤60	6	31	11	7	89.1%	55	3.271	0.352
>60	10	19	6	2	73%	37		
Pathology								
Normal	0	3	7	0	1	10	15.598	0.004*
Paracarcinoma	0	8	21	8	1	37		
Adenocarcinoma	0	4	17	24	1	45		

Previous studies indicated that MLN8237 inhibited the Wnt and Akt signaling pathways. Thus, we measured whether MLN8237 whether MLN8237 affectβ-catenin transcriptional activity by the TOP/FOR flash reporter plasimid. Decreased activity of theβ-catenin/TCF4 in MLN8237-treatde cells was detected in MGG-803, BGC-823, SGC-7901 and U87 EGFRvIII cells (P<0.05, Figure S2).

## DISCUSSION

The generation of gastric cancer is a multifactorial and complicated process. Although much has been learned concerning the genetic and biochemical bases of gastric cancer because of the difficulties to identify and validate therapeutic targets, few novel therapeutic targets have been identified [29–31]. Recently, several groups have published GWASs of gastric cancer focusing on single-nucleotide polymorphisms (SNPs), such as in a locus on chromosome 10q23 in the PLCE1 gene or in PRKAA1 at 5p13.1 [23, 24, 32]. However, few functionally important genes have been identified in the pathogenesis of gastric cancer.

In our gastric cancer eGWAS, we identified 184 genes as significantly repeatedly dysregulated with P<1.0×10^−5^ (under the Bonferroni-corrected threshold). Using GO enrichment analysis, we found that these genes were implicated in the “cell cycle” and “collagen”. However, there was no enrichment in pathway analysis. Furthermore, our network analysis demonstrated that AURKA is the pivotal gene in the study. The role of AURKA during mitosis has been defined to a large extent [33]. Furthermore, the involvement of AURKA in the cell cycle and cell division, as well as its role in other cell signaling pathways, is well established. In addition to its well-defined role in mitosis, the overexpression of AURKA and upregulation of its enzymatic activity have been linked to tumorigenesis, specifically in ovarian, prostate, esophageal, gastric, colon and breast cancers. Additionally, a specifically inhibitor of AURKA has been designed [34–39].

Results from previous studies have suggested that the overexpression of AURKA increases the level of p-GSK-3β, p-Akt1 and β-catenin and plays an important role in cell proliferation, tumor progression and metastasis [40–42]. MLN8237 is a specific inhibitor of AURKA [43]. And our study have showed that MLN8237 decreased the level of p-GSK-3β, p-Akt1 and β-catenin in our study. Additionally, MLN8237 treatment arrested the cells in the G2/M phase and suppressed the invasive ability of cells [44–47].

Chromatin is a complex of DNA and histone proteins, and the term “epigenetics” is defined to describe heritable changes in a cellular phenotype that are independent of alterations in the DNA sequence [48]. The information conveyed by epigenetic modifications plays a critical role in the regulation of all DNA-based processes, such as transcription, DNA repair and replication, and histone modification is an important part of epigenetic modification [49]. Additionally, many histone modifications are misregulated in cancer. The Wnt/β-catenin and PI3K/Akt signaling pathways play important roles in the development of cancer, and it is important to determine the relationship between the activities of the Wnt and Akt signaling pathways and histone modification [50]. Anti-epigenetic therapy that targets the Wnt/β-catenin and EGFR pathways is vital for therapy [51]. We proposed the application of FH535, LY294002, Wnt3a and EGF, and we found the relationship between the activities of the Wnt and Akt pathways and histone methylation. KDM6A and KDM6B are important demethylases that affect signaling pathways important for endoderm differentiation, such as the Wnt and Akt signaling pathways. However, the mechanism concerning the interplay between histone methylation and the two signaling pathways remains unclear.

AURKA is a crucial candidate gene for gastric cancer and is relevant to the Wnt/β-catenin and PI3K/Akt signaling pathways. We suspected that AURKA regulates modification through the effects on the Wnt/β-catenin and PI3K/Akt signaling pathways. The results above showed that MLN8237 increased the expression of H3K27 me2/me3/AC in the gastric cancer cell lines MGC-803 and BGC-823, and SGC-7901 decreased the expression of H3K4 me1/me3 and H3K27 me1/me3 in U87 EGFRvIII cells. According to previous studies, H3K4 is associated with active genes in euchromatin, whereas H3K27 is associated with heterochromatic regions of the genome. Histone methylation plays an important role in diverse biological processes, including transcription, and can function in both gene activation and repression [14–16]. Additionally, different methylation states on the same residue can also localize differently. H3K4 me2/3 usually spans the transcriptional start site of active genes, whereas H3K4 me1 is a modification associated with active enhancers, and trimethylation of H3K27 is associated with gene repression [52].

Twist is a crucial gene in the development of the embryo and stimulate the process of EMT [53]. Epigenetic silencing has been studied as a potential candidate for targeted therapy. ChIP analysis showed that the level of H3K27 me3 in the promoter of Twist increased following MLN8237 treatment, and we suspect that epigenetic regulation was an approach of MLN8237 function. And MLN8237 decreased Twist and inhibited EMT. A number of prior studies have suggested that EMT is associated with increased resistance of cancer cells to anticancer drugs [54]. But statistically significant positive correlations between expression levels of the epithelial biomarker E-cadherin and drug sensitivity was found for only ten out of 118 drugs [55]. We need more efforts to research whether inhibition of AURKA could damage gastric cancer's drug resistance by reverse EMT. The blocking of AURKA suppressed the activity of the two signaling pathways and inhibited the expression of EMT-related genes, including Twist, through regulating histone modification. As we all know, AURKA is not only correlated with the Wnt and Akt signaling pathways but also may regulate histone modification through other ways. Additionally, the mechanism underlying the effect on histone modification by AURKA was not investigated, and we need to identify more downstream genes related to AURKA to clarify the effect of AURKA on gastric cancer. In conclusion, the study provides insights into the relevance of AURKA and related genes for the progression of EMT, reveals the significance of histone modification in the progression and highlights their potential to be exploited as therapeutic targets.

## MATERIALS AND METHODS

### eGWAS

All of the gastric cancer-related, genome-wide microarray experiments used in this study were collected from the NCBI Gene Expression Omnibus (GEO, www.ncbi.nlm.mih.gov/geo) public database. There were 679 samples (428 gastric cancer cases and 251 controls) in 13 independent databases. To estimate differences between the groups of samples from diabetic subjects and groups representing the control, raw post quantitation microarray data were reanalyzed using Significance Analysis of Microarrays software (SAM). There are 30,663 genes in the database. For each gene, we counted the observed number of microarray experiments in which the gene was significantly dysregulated. We next calculated *P* values from the number of positive/negative experiments for each gene and sums of the number of positive/negative experiments for all of the other genes using Fisher's exact test as an alternative. The Bonferroni threshold (*P*=1.0×10^−5^) indicated that there were 184 gastric cancer susceptibility genes.

We applied these 184 genes to the gene ontology database, and divided them into the following three parts: biological process, cellular component, molecular function through GSEABase. Next, we performed enrichment analysis across DAVID online tools. Furthermore, we applied these genes to the KEGG pathway database through GneMAPP v2.1 and calculated the enrichment *P*-value. Finally, we performed network analysis across medusa, including the KEGG database, experiments and the literature.

### Cell culture

The human gastric cancer cell linesMGC-803, BGC-823, and SGC-7901, hepatocarcinomacell line Hep-3B, colon cancer cell line (Caco2) and glioblastoma cell lineU87 were obtained from the China Academia Sinica Cell Repository, Shanghai, China. The human glioblastoma cell line U87 EGFRvIII was donated by the Laboratory of Neuro-oncology, Tianjin Neurological Institute (Tianjin, China). U87vlll cells carry a truncated mutant EGFR gene, which can be activated without EGF stimulation consistently [19]. Cells were cultured in DMED/1640 medium supplemented with 10% FBS and incubated at 37°C in 5% CO_2_.

### Chemical reagents and antibodies

For *in vitro* studies, stock solutions of MLN8237 (20 mmol/L), FH535 (10 mmol/L) and LY294002 (20 mmol/L) were prepared in DMSO. Stock solutions of recombinant human Wnt3a (100 μg/ml), EGF (100 μg/ml) and TGF-β1(100 μg/ml) were diluted in phosphate-buffered saline (PBS).

Dimethyl sulfoxide (DMSO), LY294002 and EGF were purchased from Sigma. FH535 was purchased from Merck. Wnt3a was purchased from abnova. MLN8237 was purchased from selleck.

AURKA, β-catenin, AKT1, p-AKT1, GSK-3β, p- GSK-3β, Twist, H3K4 me3/AC and H3K27 me2/me3/AC antibodies were purchased from Abcam. E-cadherin, N-cadherin, H3K4 me1/me2 and H3K27 me1 antibodies were purchased from Cell Signaling Technology. The Ki67 antibody was purchased from Santa Cruz Biotechnology. The H3 antibody was purchased from Ray Antibody. The GAPDH antibody was purchased from Zhongshan Bio Corp.

### Western blot analysis

The cells were harvested at 24 h after the treatment of MLN8237, and the total protein was extracted using RIPA lysis buffer containing proteinase inhibitors. The homogenates were clarified by centrifugation at 12,000×g for 15 min at 4°C. Nuclear protein was extracted using the Nuclear and Cytoplasmic Protein Extraction Kit (Beyotime Biotechnology), according to the manufacturer's protocols. Next, the protein concentration was measured using the bicinchoninic acid method. A total of 40μg of protein mixed with 4×SDS loading buffer was loaded into each lane and separated by 10% or 15% sodium dodecyl sulfate-polyacrylamide gel electrophoresis (SDS-PAGE). The separated proteins were transferred to PVDF membranes (Millipore), and the membranes were incubated in the above described primary antibodies, followed by incubation with HRP-conjugated secondary antibody (Zhongshan Bio Corp, Beijing, China). Protein expression was visualized using the SuperSignal protein detection kit (Pierce, USA). The membrane was stripped and reprobed with Histone H3 (Ruikang, 1:2000 dilution), GAPDH primary antibody (Zhongshan Bio Corp, Beijing, China) as a control.

### Cell cycle analysis

The effect of MLN8237 on cell cycle distribution was determined by flow cytometry. Briefly, MGC-803, BGC-823, SGC-7901 and U87 EGFRvIII were treated with MLN8237 at 10μM for 24 hours. Cells in the growth log phase were harvested, washed with PBS, fixed with 70%ethanol overnight at 4°C, and then incubated with RNase at 37°C for 30 min. The cell nuclei were stained with propidium iodide for an additional 30 min. Cells were examined in a flowcytometer, and the results are presented as the percentage of cells in each phase.

### Transwell invasion assay

The ability of cells to migrate was assessed using matrigel-coated transwell membranes (Becton-Dickinson). After 30 min of incubation at 37°C, the matrigel solidified and served as an extracellular matrix for tumor cell invasion analysis. The cells were then incubated for 48 hours at 37°C in 5% CO_2_. Cell invasion to the underside of the matrigel-coated membrane was detected by staining cells with crystal violet, counting them and then imaging them under an inverted microscope at ×200 magnification (Olympus Corp, Tokyo, Japan). The results were expressed as the average number of invasive cells per field.

### Chromatin immunoprecipitation (ChIP)

To test whether there was H3K27 me3 bound to the promoter of Twist, cells were treated with DMSO or MLN8237 for 24 hours prior to formaldehyde fixation. Next, cells were harvested for chromatin immunoprecipitation (ChIP) using the EZ-ChIP kit (Millipore), according to the manufacturer's protocols. Chromatin was extracted, and cross-linked DNA was cut to approximately 200-1000 base pairs. Protein G Agarose was added to the antibody/chromatin complexes and incubated overnight at 4°C. H3K27 me3 antibodies were used to pull down DNA from formaldehyde cross-linked chromatin. The protein G Agarose-antibody/chromatin complex was resuspended in wash buffer and centrifuged to collect the protein/DNA complex. The protein/DNA cross-links were reversed to obtain free DNA. Purified DNA was quantified using real-time quantitative PCR.

### Animal experiments

BALB/c-A nude mice at 4 weeks of age for animal experiments were purchased from the Animal Center of the Cancer Institute, Chinese Academy of Medical Science. We estimated the therapeutic potential of MLN8237 using xenograft models and MGC-803 gastric cancer cells. Additionally, the mice were randomly assigned to four groups (six subcutaneous tumors/group). In Group 1, 100μL of DMSO/DMEM (1:1) was intraperitoneally injected into the xenograft tumor model. In Group 2, MLN8237 (20 mg/kg/dose) dissolved in 100 ml DMSO/DMEM was intraperitoneally injected every 2 days during the experimental period.

To test the effect of knocking down AURKA, lentiviruses containing an AURKA inhibitor sequence (Lenti-siAURKA) or negative control (Lenti-NC) were obtained from Shanghai Genechem, China. Additionally, 2×10^6^ MGC-803 cells and 10^5^ U87 EGFRvIII cells stably expressing Lenti-siAURKA were injected into the peritoneal cavity, and the brain underwent intraperitoneal and intracranial inoculation. The animal groups were as follows: group 3: MGC-803 cells expressing Lenti-NC; group 4: MGC-803 cells expressing Lenti-siAURKA; group 5: U87 EGFRvIII cells expressing Lenti-NC; group 6: U87 EGFRvIII cells expressing Lenti-siAURKA. The tumor volumes were quantified using calipers to measure the tumor length and width. The weight of the mice was measured every 2 days, and the tumor weight was surveyed at the endpoint of the study. Mice in groups 5 and 6 were imaged for Fluc activity using bioluminescence imaging as described previously [20]. The weight of mice was measured every 2 days.

### Immunohistochemistry staining

Tissue microarrays were purchased from Alenabio, and the specimens were classified according to World Health Organization (WHO) categories (2007) under an institutional review board. Samples included 6cases of WHO Grade I gastric adenocarcinoma, 30 cases of WHO Grade II gastric adenocarcinoma, 9 cases of WHO Grade III gastric adenocarcinoma and 55 cases of normal gastric mucosa.

For immunohistochemical staining, formalin-fixed tissue samples were prepared as paraffin-embedded sections, and immunostaining was performed on sections using the avidin-biotin-complex method. Primary antibodies specific for H3K27 me3 (Abcam; 1:100 dilution) and Ki67 (Santa Cruz; 1:100 dilution) were diluted in PBS with 0.1% Tween 20 and incubated overnight at 4°C. The sections were incubated in biotinylated secondary antibodies (Zhongshan Biology; 1:100 dilution) at 37°C for 1 hour, followed by incubation in avidin-biotin complex solution for an additional 1 hour. Protein expression was detected by coloration with diaminobenzidine (DAB) buffer, and the sections were counterstained with hematoxylin. Staining results were scored by two pathologists. Sections with no labeling, or with <5% labeled cells, were score as 0. Sections with 5-30% of positive cells were scored as 1, sections with 31-70% of positive cells were scored as 2, and sections with≥71% of positive cells were scored as 3. The staining intensity was scored similarly, as described previously [21].

### Statistical analysis

The significance of Kaplan-Meier statistics was tested using the log-rank test. Multivariate analysis was performed using the multivariate Cox regression model. SPSS 16.0 (SPSS, Chicago, IL) was used for all of the calculations. All of the data were represented by the mean ± SD. Statistical significance was determined at *P*<0.05.

## SUPPLEMENTARY TABLES AND FIGURES






