# Supplementary material for: AURKA induces EMT by regulating histone modification through Wnt/β-catenin and PI3K/Akt signaling pathway in gastric cancer
Source: Oncotarget. 2016 Apr 21;7(22):33152–64. doi: 10.18632/oncotarget.8888 (PMC5078082; doi:10.18632/oncotarget.8888)
Supplement: Supplementary file 1 [file oncotarget-07-33152-s001.pdf]

SUPPLEMENTARY TABLES AND FIGURES

Supplementary Table S1: The highest ranked 184 genes in gastric cancer eGWAS

See Supplementary File 1

Supplementary Table S2: KEGG pathway analysis and calculated the enrichment P-value

See Supplementary File 2

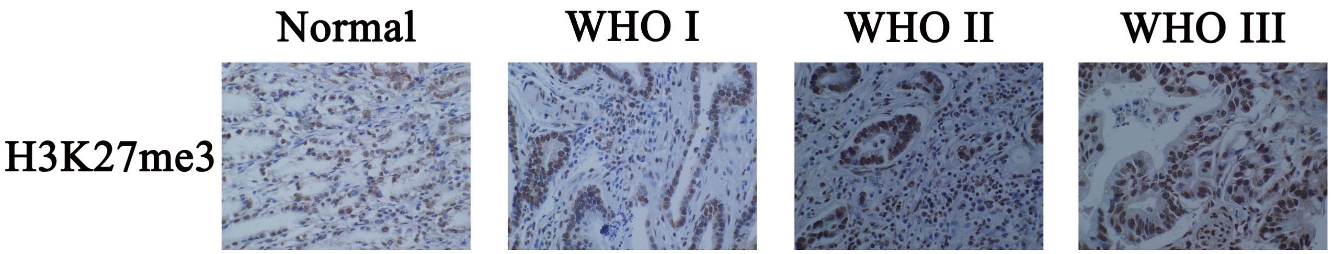

Supplementary Figure S1: Expression of H3K27me3 is detected in gastric cancer.

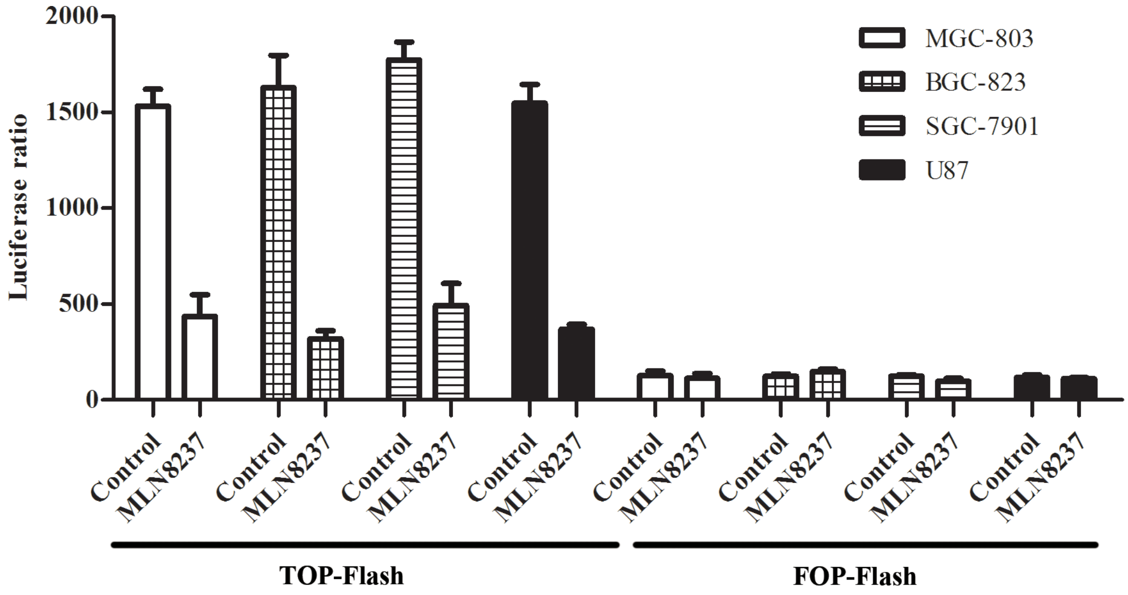

Supplementary Figure S2: The activity of Akt and Wnt signaling pathways were measured by dual-luciferase reporter assays.

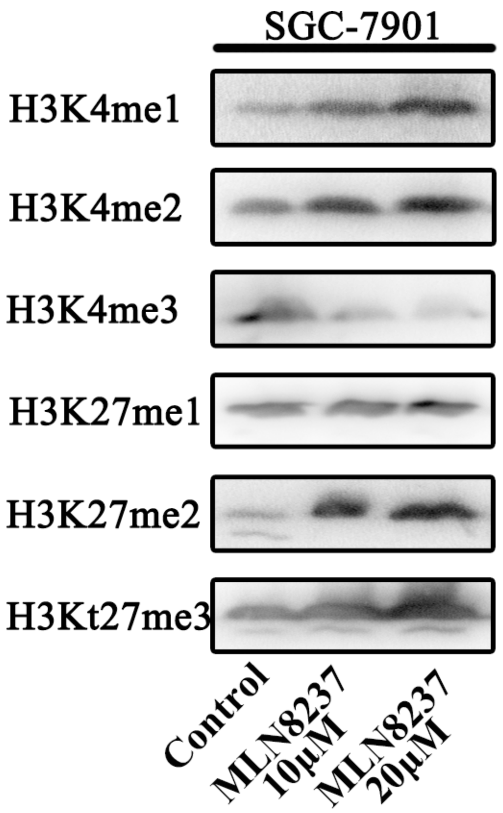

Supplementary Figure S3: The change of H3K4me1/2 and H3K27me3 were concentration-dependent after the treatment of MLN8237.
